# q2-metnet: QIIME2 package to analyse 16S rRNA data via high-quality metabolic reconstructions of the human gut microbiota

**DOI:** 10.1093/bioinformatics/btae455

**Published:** 2024-07-17

**Authors:** Francesco Balzerani, Telmo Blasco, Sergio Pérez-Burillo, M Pilar Francino, José Á Rufián-Henares, Luis V Valcarcel, Francisco J Planes

**Affiliations:** Tecnun School of Engineering, Biomedical Engineering and Sciences Department, University of Navarra, San Sebastian 20018, Spain; Tecnun School of Engineering, Biomedical Engineering and Sciences Department, University of Navarra, San Sebastian 20018, Spain; Tecnun School of Engineering, Biomedical Engineering and Sciences Department, University of Navarra, San Sebastian 20018, Spain; Area de Genómica y Salud, Fundación para el Fomento de la Investigación Sanitaria y Biomédica de la Comunitat Valenciana-Salud Pública, Valencia, 46020, Spain; CIBER en Epidemiología y Salud Pública, Madrid, Spain; Departamento de Nutrición y Bromatología, Instituto de Nutrición y Tecnología de los Alimentos, Centro de Investigación Biomédica, Universidad de Granada, Granada, 18071, Spain; Instituto de Investigación Biosanitaria ibs.GRANADA, Universidad de Granada, Granada, 18071, Spain; Tecnun School of Engineering, Biomedical Engineering and Sciences Department, University of Navarra, San Sebastian 20018, Spain; Biomedical Engineering Center, University of Navarra, Pamplona 31009, Spain; Instituto de Ciencia de los Datos e Inteligencia Artificial (DATAI), University of Navarra, Pamplona 31009, Spain; Tecnun School of Engineering, Biomedical Engineering and Sciences Department, University of Navarra, San Sebastian 20018, Spain; Biomedical Engineering Center, University of Navarra, Pamplona 31009, Spain; Instituto de Ciencia de los Datos e Inteligencia Artificial (DATAI), University of Navarra, Pamplona 31009, Spain

## Abstract

**Motivation:**

16S rRNA gene sequencing is the most frequent approach for the characterization of the human gut microbiota. Despite different efforts in the literature, the inference of functional and metabolic interpretations from 16S rRNA gene sequencing data is still a challenging task. High-quality metabolic reconstructions of the human gut microbiota, such as AGORA and AGREDA, constitute a curated resource to improve functional inference from 16S rRNA data, but they are not typically integrated into standard bioinformatics tools.

**Results:**

Here, we present q2-metnet, a QIIME2 plugin that enables the contextualization of 16S rRNA gene sequencing data into AGORA and AGREDA. In particular, based on relative abundances of taxa, *q2-metnet* determines normalized activity scores for the reactions and subsystems involved in the selected metabolic reconstruction. Using these scores, q2-metnet allows the user to conduct differential activity analysis for reactions and subsystems, as well as exploratory analysis using PCA and hierarchical clustering. We apply q2-metnet to a dataset from our group that involves 16S rRNA data from stool samples from lean, allergic to cow’s milk, obese and celiac children, and the Belgian Flemish Gut Flora Project cohort, which includes faecal 16S rRNA data from obese and normal-weight adult individuals. In the first case, q2-metnet outperforms existing algorithms in separating different clinical conditions based on predicted pathway abundances and subsystem scores. In the second case, q2-metnet complements competing approaches in predicting functional alterations in the gut microbiota of obese individuals. Overall, q2-metnet constitutes a powerful bioinformatics tool to provide metabolic context to 16S rRNA data from the human gut microbiota.

**Availability and implementation:**

Python code of q2-metnet is available in https://github.com/PlanesLab/q2-metnet and https://figshare.com/articles/dataset/q2-metnet_package/26180446.

## 1 Introduction

Thanks to the development of new sequencing technologies and computational methods (Callahan *et al.* 2016, Nguyen *et al.* 2016, Bokulich *et al.* 2018), we have gained a deeper insight into the human gut microbiota, whose key role in health and disease has been firmly established (Rowland *et al.* 2018, Pérez-Burillo *et al.* 2021). 16S rRNA gene sequencing is the most frequent approach for the characterization of the human gut microbiota. High-quality bioinformatic tools, such as QIIME2 (Bolyen *et al.* 2019), are freely available and allow researchers to easily analyse and visualize 16S rRNA gene sequencing data.

However, the metabolic functions of bacterial communities cannot be directly extracted from 16S rRNA gene sequencing data. Different computational methods have been derived to address this issue, including PiCRUSt2 (Douglas *et al.* 2020), Tax4Fun2 (Wemheuer *et al.* 2020), or MicFunPred (Mongad *et al.* 2021), among others. In essence, these algorithms infer gene abundance based on 16S rRNA gene sequences and predict pathway abundances with standard metabolic databases, such as KEGG (Kanehisa and Goto 2000, Kanehisa *et al.* 2016) or MetaCyC (Caspi *et al.* 2018).

In the case of the human gut microbiota, the release of large-scale metabolic models, such as AGORA (Magnúsdóttir *et al.* 2017, Heinken *et al.* 2023) or AGREDA (Blasco *et al.* 2021, Balzerani *et al.* 2022), create new possibilities to provide metabolic context to 16S rRNA gene sequencing data. These metabolic models integrate high-quality genome-scale metabolic reconstructions for hundreds of organisms present in the human gut, including annotated reactions, metabolites, and pathways. While these metabolic models have been previously used to simulate the behaviour of bacterial communities via constraint-based modelling (Bauer and Thiele 2018, Heinken *et al.* 2021), they have been little exploited for standard bioinformatics pathway analysis of 16S rRNA gene sequencing data. Large-scale metabolic models can provide a reaction-centric strategy that could complement existing metagenomic approaches (Douglas *et al.* 2020, Wemheuer *et al.* 2020, Mongad *et al.* 2021), based on standard metabolic databases (Kanehisa and Goto 2000, Kanehisa *et al.* 2016, Caspi *et al.* 2018), which incorporate limited information about transport reactions from diet, for example. mgPipe (Baldini *et al.* 2019), a computational module of the Microbiome Modeling Toolbox (Heinken and Thiele 2022), among different functionalities, enables this type of reaction centric functional analysis for 16S rRNA gene sequencing data. However, mgPipe is available in MATLAB and not in QIIME2, which limits their scope of application for human gut microbiome studies. Moreover, the performance of mgPipe in comparison with other pathway analysis tools has not been assessed in detail.

Here, we present q2-metnet, a QIIME2 package able to contextualize taxonomic abundance data into high-quality metabolic models of the human gut microbiota. q2-metnet provides functional context to 16S rRNA gene-sequencing data, following a reaction centric approach that differs from mgPipe and existing gene-centric bioinformatic tools. The article is organized as follows. First, we describe the mathematical approach implemented in q2-metnet to quantify the activity of reactions and pathways based on 16S rRNA gene-sequencing data, as well as the subsequent statistical and exploratory analysis. Second, we illustrate the proposed approach by means of a toy example. In addition, we apply q2-metnet to: (i) a previously published dataset from our group, which involves 16S rRNA gene sequencing data from stool samples from lean, allergic to cow’s milk, obese, and celiac children; and (ii) the Belgian Flemish Gut Flora Project (BFGFP) cohort (Falony *et al.* 2016), which enables a large-scale comparison of faecal 16S rRNA gene sequencing data from obese and normal-weight individuals. Using these datasets, we compare q2-metnet with previous methods to infer the activity of metabolic pathways with 16S rRNA gene sequencing data, particularly mgPipe, PiCRUSt2, Tax4Fun2, and MicFunPred.

## 2 Materials and methods

q2-metnet is a python package that has been integrated within QIIME2 (Bolyen *et al.* 2019), a popular bioinformatic tool for the analysis of microbiome data. The main function, q2-metnet.generateFeatures, extracts a normalized activity score for every reaction present in the selected metabolic reconstruction across the different samples under study, according to their taxonomic composition and relative abundance obtained from 16S rRNA gene sequencing data. These scores are scaled from 0 to 1, which allows us to compare the potential presence of these reactions under different conditions. Once we have the activity score of reactions, we proceed to determine the scores for the different metabolic subsystems. We describe below the systematic procedure to calculate these normalized activity scores for reactions and subsystems.

### 2.1 Calculation of normalized activity score of reactions and subsystems

**Figure 1.**
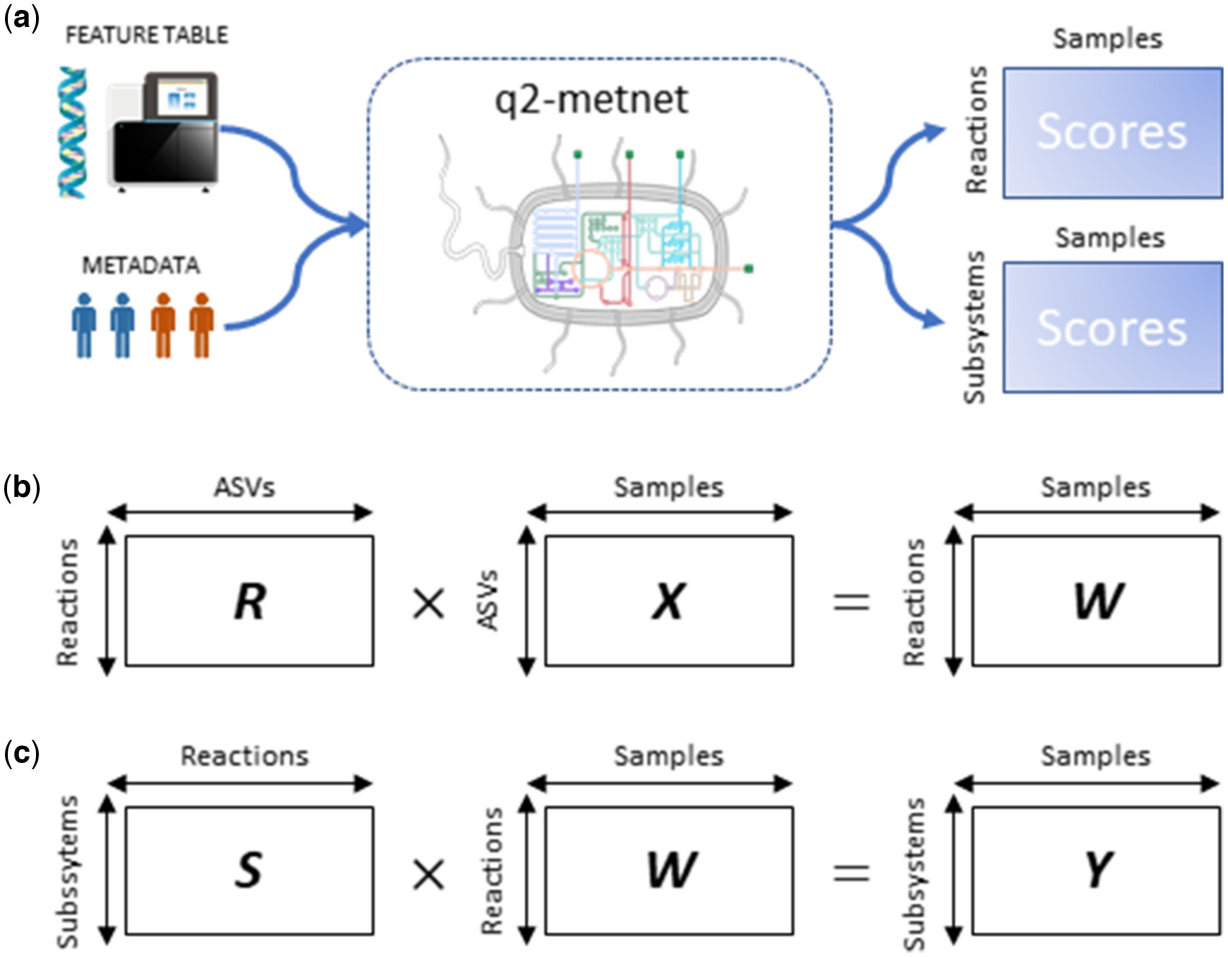
q2-metnet procedure to calculate the normalized activity scores of reactions and subsystems. (a) Representation of input and output data of the main function of the package: generateFeatures. The input data for this function is the abundances of taxa in the samples (feature table), and sample metadata. An output table with the normalized activity scores of reactions and subsystems for the different samples is generated. The user can select among different metabolic reconstructions and taxonomic category to calculate these normalized scores. (b) Calculation of matrix *W*, which stores the normalized activity scores of reactions per sample. Matrix *X* stores the relative frequencies of ASVs per sample, whereas matrix *R* provides a presence ratio of reactions in identified ASVs. (c) Calculation of matrix *Y*, which stores the normalized activity scores of subsystems per sample. Matrix *S* provides the relative contribution of reactions to subsystems. *Source*: https://bioicons.com/.

The input data for the calculation of normalized activity scores are: (i) sample taxo0nomic abundances (feature table); and (ii) sample metadata (Fig. 1a). Feature tables include counts of taxonomic features, such as ASVs (Amplicon Sequence Variant) or OTUs (Operational Taxonomic Unit), for the different samples. q2-metnet can employ taxonomy feature tables generated from 16S rRNA gene sequences using the standard taxonomy assignation tools within QIIME2 (e.g. Naive Bayes taxonomic classifier with Silva138 database), or else by means of any other external tools if preferred by the user. Moreover, the user can modify two parameters: (i) the metabolic reconstruction: AGREDA version 1.0.0 (default), AGORA, version 1.0.3, and AGORA2, version 2.0.1, which defines the reactions involved in the different taxa; (ii) taxonomic category, ranging from genus to species (default) level. Taxa not available in the selected reconstruction were not considered in our analysis. We describe below the different steps for the calculation of normalized activity scores.

**Step 1: Feature table normalization and network contextualization**. For each sample, we only consider ASVs in the feature table with an unambiguous assignation at the taxonomic category selected by the user. Entries that point to the same ASV are merged, and their counts added up. Next, the resulting feature table for each sample is converted into relative frequencies, namely by dividing each ASV count by the total sum of counts. Therefore, the sum of the relative ASV frequencies per sample is equal to 1.

Then, q2-metnet automatically searches for ASVs within the taxa in the selected reconstruction: 818 in both AGREDA and AGORA, while 7302 in AGORA2 (Supplementary Table S1). ASVs without any association to the taxa in the selected reconstruction are deleted, leading to matrix X of dimensions *u* (number of ASVs) and *n* (number of samples) (Fig. 1b), which stores the relative abundance of ASVs with matching taxa in the selected reconstruction for different samples. Note here that although metabolic reconstructions typically work at the strain level, the taxonomical assignation of 16S rRNA data usually reaches the species level at most. Thus, different strains could be assigned to an identified ASV. This is considered in Step 2.

**Step 2: Reaction-ASV matrix definition**. Once we have the list of *u* ASVs with a matching strain at the genus or species level in the selected metabolic reconstruction, we define the matrix *R* of dimensions *r* (number of reactions) and *u* (number of ASVs), which associates each reaction in the selected reconstruction with every matching ASV (Fig. 1b). To that end, we first check the presence of every reaction *k* (*k *=* *1, …, *r*) in any of the strains associated to the ASV *i* (*i *=* *1, …, *u*). In the positive case, *R_ki_* will be the ratio between the number of occurrences of reaction *k* in the strains associated with ASV *i* and the total number of strains associated with ASV *i*. For example, for an ASV *i* with four associated strains, if the reaction *k* is present in three out of four strains, *R_ik_* = 0.75. In the negative case, *R_ki_* = 0. This strategy ensures that the reaction is given full weight (*R_ki_* = 1) when it is present in all strains of the genus or species and a lower weight when it is present only in a subset of them. Note here that mgPipe always provides full weight (*R_ki_* = 1) even if the reaction is not present in all of the strains of the genus or species identified.

In addition, the list of reactions also includes input/output metabolite exchanges (transport reactions). Input exchanges define the capacity of a particular ASV to degrade nutrients from the diet, while output exchanges define the release of microbial metabolites. Supplementary Table S2 details the set of reactions available in the different metabolic models of available human gut microbiota reconstructions.

**Step 3: Reaction-Sample score matrix definition**. Once *X* and *R* have been determined, we calculate the normalized activity score for each reaction and sample with the following matrix product (Fig. 1b):

(1) W=R·X 

Reaction activity score *W_kj_* represents the presence ratio of reaction *k* in sample *j* considering the relative frequency of identified ASVs.

**Step 4: Subsystem-Reaction matrix definition**. In order to obtain the normalized activity scores for subsystems, we define the matrix *S* of dimensions *s* (number of subsystems) and *r* (number of reactions), which associates reactions to the different subsystems (Fig. 1c). To that end, we first check the presence of every reaction *k* (*k *=* *1, …, *r*) in any of the subsystems *z* (*z *=* *1, …, *s*). In the positive case, *S_zk_* will be 1 over the total number of reactions annotated to subsystem *z*. For example, for a subsystem *z* with 10 reactions, the score for each of these reactions *k* is *S_zk_* = 1/10. In the negative case, *S_zk_* = 0. Consequently, the sum of the normalized reaction values per subsystem is equal to 1. In contrast with q2-metnet, mgPipe contextualizes matrix *S* for each sample, namely by only considering the number of reactions of a subsystem that are present in the sample. For illustration, in the above example, if a specific sample includes 6 out of 10 reactions, then mgPipe would set *S_zk_* = 1/6 instead of *S_zk_* = 1/10.

Note here that subsystems constitute annotated metabolic functions in the selected reconstruction. However, we manually classified exchange reactions into different families of metabolites, deriving up to 57 additional subsystems (Supplementary Table S3), so-called compound exchange (CE) subsystems. These new subsystems can be very useful to analyse the capabilities of the human gut microbiota to degrade input nutrients or produce output microbial metabolites.

**Step 5: Subsystem-Sample score matrix definition**. Once *S* and *W* have been determined, we calculate the normalized activity score for each subsystem and sample with the following matrix product (Fig. 1c):

(2) Y=S·W

The subsystem activity score *Y_zj_* represents the presence ratio of subsystem *z* in sample *j* considering the relative frequency of identified ASVs and associated reactions.

### 2.2 Statistical and exploratory analysis in *q2-metnet*

Once we have calculated the matrices *W* and *Y*, q2-metnet permits to conduct a differential activity analysis of reactions and subsystems for the different conditions using available metadata of samples. In particular, we make use of a Wilcoxon unpaired two-sample test to assess mean differences in reaction and subsystem activity. *P*-values are adjusted for multiple hypothesis testing with the False Discovery Rate (FDR) approach. Significant differences can be visualized via boxplots. Moreover, q2-metnet allows the user to conduct Principal Component Analysis (PCA) or a hierarchically-clustered heatmap to visualize the reaction and subsystem activity score of different samples in a low dimensional space.

## 3 Results

### 3.1 Illustrative example of the *q2-metnet* approach

**Figure 2.**
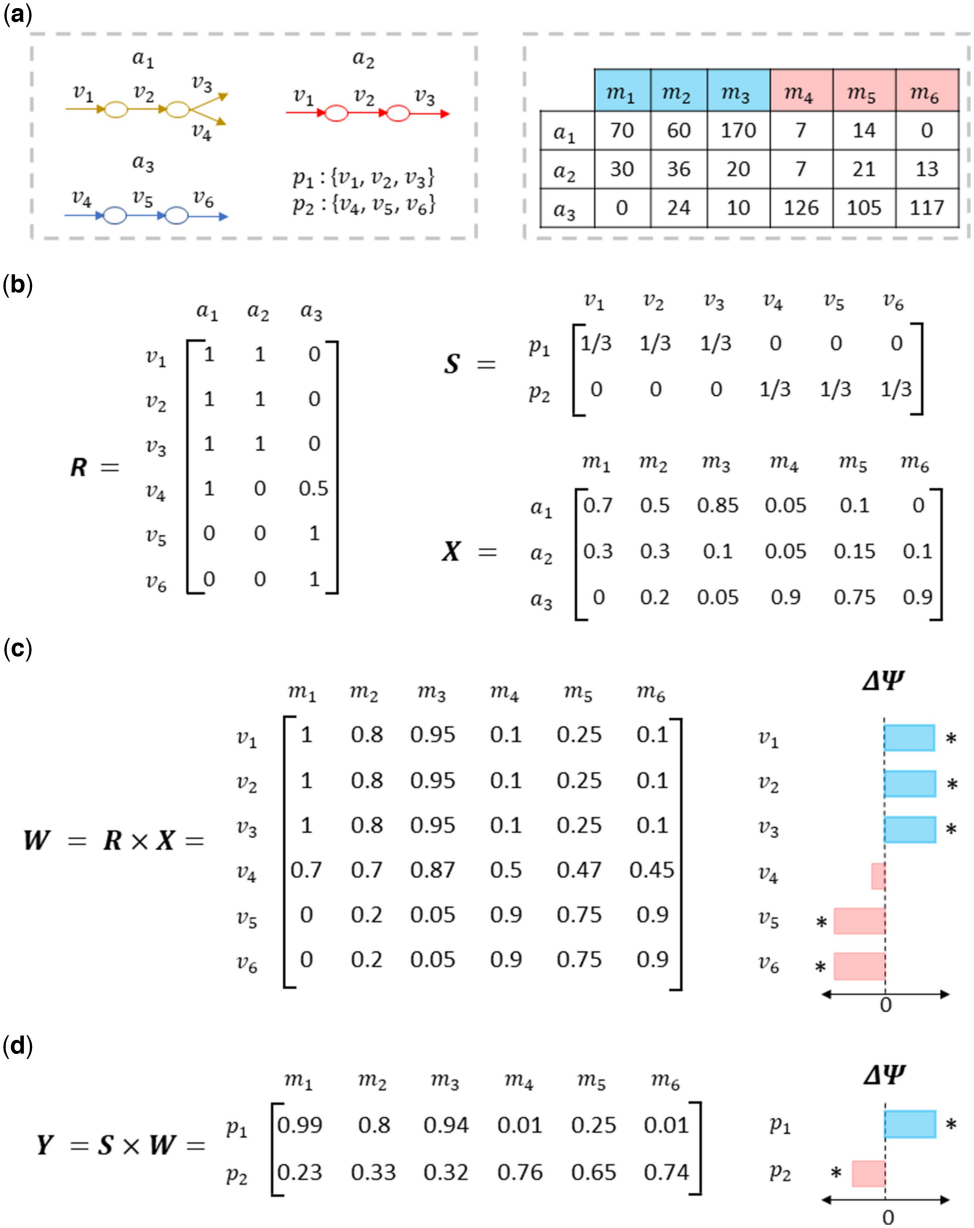
Illustrative example of the q2-metnet computational approach. (a) Input data for the q2-metnet algorithm. On the left side, the metabolic models of each example ASV $a_{i}$ (*i *=* *1, 2, 3) identified in the microbial communities are shown, where $v_{k}$ (*k *=* *1, …, 6) and $p_{s}$ (*s *=* *1, 2) represent their associated metabolic reactions and subsystems, respectively. On the right side, an example of microbial abundance data is shown, which involves six samples ($m_{j}$, *j *=* *1, …, 6): three controls (m_1_, m_2_, m_3_) and three test samples (m_4_, m_5_, m_6_); (b) Resulting matrices R, S, and X obtained from input data; (c) Calculation of matrix W and differential activity analysis for reactions; (d) Calculation of matrix Y and differential activity analysis for subsystems. Note that (*) denote statistical significance (Wilcoxon adjusted *P*-value <.05).

We present in Fig. 2a complete toy example involving three identified ASVs (*a*_1_, *a*_2_, *a*_3_) in six different samples (*m*_1_, *m*_2_, *m*_3_, *m*_4_, *m*_5_, *m*_6_), which are associated with two conditions of interest. In addition, once ASVs are assigned to metabolic models in the selected reconstruction, we assume six reactions (*v*_1_, *v*_2_, *v*_3_, *v*_4_, *v*_5_, *v*_6_) and two subsystems (*p*_1_, *p*_2_). Figure 2a shows the input data required to run q2-metnet, namely the feature table derived from 16S rRNA sequencing analysis, and the reactions associated to different ASVs and metabolic subsystems according to the selected reconstruction. As detailed in the Section 2, input data is internally organized into three matrices: *X*, *R*, and *S* (Fig. 2b). First, matrix X is extracted by calculating the relative microbial composition of each sample. For example, in the first sample (*m*_1_), the relative abundances of the first, second, and third ASVs (*a*_1_, *a*_2_, *a*_3_) are 0.7, 0.3, and 0, respectively. Second, matrix *R* assumes that every reaction present in a particular ASV takes part in all its associated metabolic models (*R_ik_* = 1), except for reaction 4 (*v*_4_) in the third ASV (*a*_3_), which it is only involved in 50% of the models (*R*_43_ = 0.5). Finally, the S matrix is defined as the contribution of each reaction to every subsystem, e.g. the first subsystem comprises the first three reactions: *S*_11_ = *S*_12_ = *S*_13_ = 1/3 and *S*_14_ = *S*_15_ = *S*_16_ = 0.

Once the matrices *X*, *R*, and *S* are defined, we proceed to calculate the normalized activity scores for reactions and subsystems (Fig. 2c and d). On the one hand, the reaction activity scores are stored in matrix W, which is calculated by the product of R with X. On the other hand, the subsystem activity scores are stored in matrix Y, being defined as the product of S with W. Finally, a differential activity analysis between different groups of samples can be performed for both reactions and subsystems (Fig. 2c and d). By default, q2-metnet makes use of the Wilcoxon test to evaluate these differences. P-values are corrected for multiple hypothesis testing using the FDR approach. Additionally, a measurement of the effect size (ΔΨ) is calculated as the difference of the mean activity scores between sample groups.

### 3.2 Metabolic analysis of children’s faeces from different clinical conditions

To illustrate q2-metnet, we analysed a previously published dataset from our group (Pérez-Burillo *et al.* 2021), which involves 16S rRNA gene-sequencing data from stool samples from one healthy lean child, one child allergic to cow’s milk, one obese child, and one celiac child. In that study, fresh faecal samples were *in vitro* fermented on lentils over 6 days, resulting in seven measurements of microbial composition for each child (6 days plus initial sample), excepting the lean child for whom there were only six samples. The taxonomic assignment of samples was done using the same strategy as in that work. In brief, we applied DADA2 algorithm (Callahan *et al.* 2016) to assign ASVs at 100% identity. Then, for those ASVs that were not annotated with DADA2, we applied the MegaBLAST module from BLAST (Altschul *et al.* 1990), requiring a unique match with at least 97% identity. Then, we applied the function generateFeatures under the default parameters: AGREDA metabolic reconstruction and species-level taxonomic assignment. We found in AGREDA 87 unique taxa out of the 141 identified species in the dataset and extracted the normalized score for each reaction and subsystem for the different samples (Supplementary Table S4).

**Figure 3.**
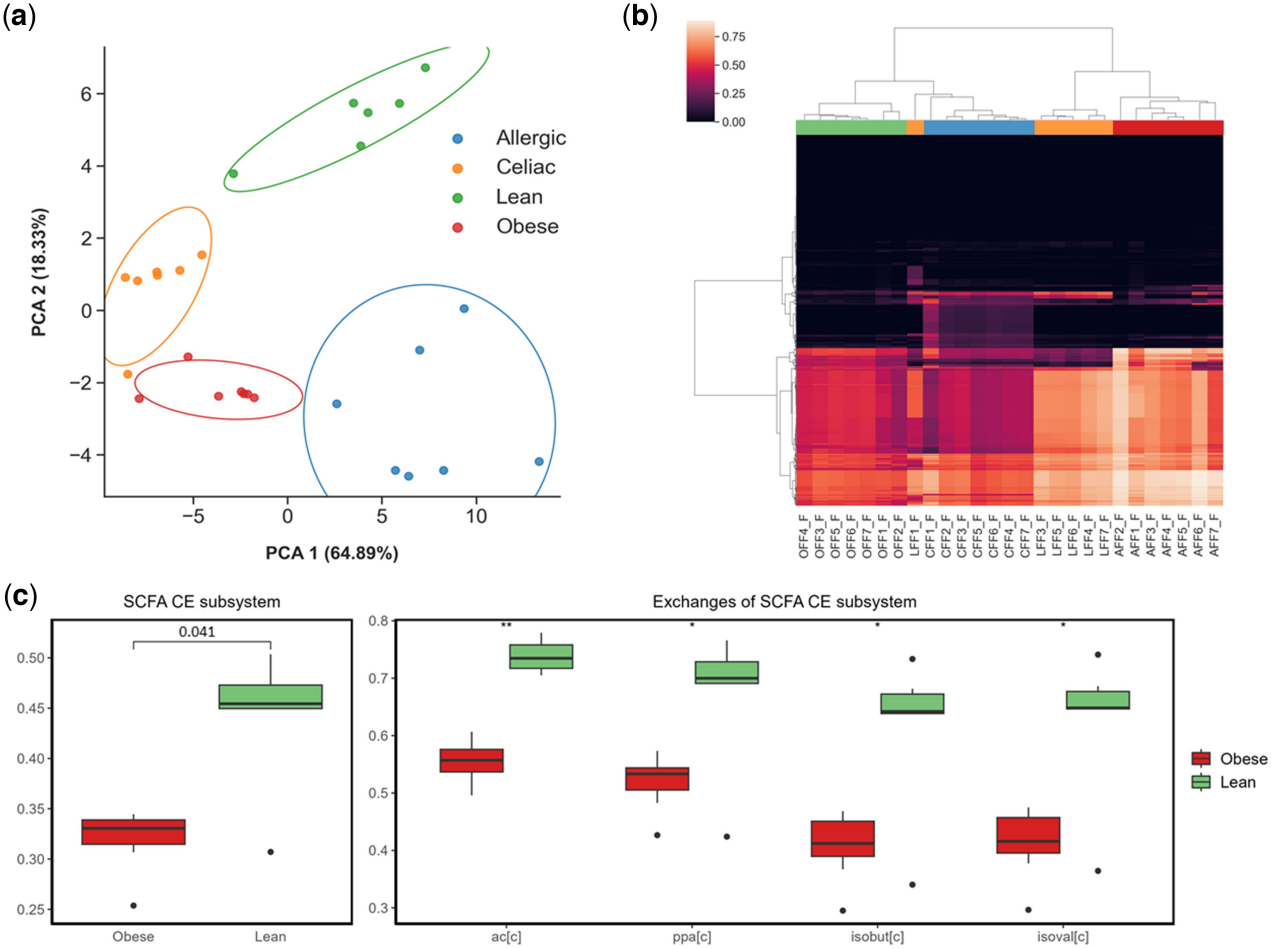
Case study of q2-metnet. (a) Principal component analysis (PCA) on reaction scores to differentiate the four individuals (lean, allergic to cow’s milk, obese, and celiac). The ellipses are generated considering the covariance and standard deviation of the data for each individual. (b) Hierarchically-clustered heatmap on the whole set of reactions present in the metabolic reconstruction. Colour bars below the dendrogram correspond to the four individuals: allergic, celiac, lean, and obese. (c) Boxplot of activity score of the SCFA CE subsystem and its corresponding exchange reactions in obese and lean children. *P*-value was calculated using two-sample Wilcoxon test and adjusted with the False Discovery Rate approach. ‘AFF2’, ‘AFF3’, ‘AFF4’, ‘AFF5’, ‘AFF6’, and ‘AFF7’ denote samples 2, 3, 4, 5, 6, and 7 from the child allergic to cow’s milk, respectively; ‘CFF1’, ‘CFF2’, ‘CFF3’, ‘CFF4’, ‘CFF5’, ‘CFF6’, and ‘CFF7’ denote samples 1, 2, 3, 4, 5, 6, and 7 from the celiac child, respectively; ‘LFF2’, ‘LFF3’, ‘LFF4’, and ‘LFF6’ denote samples 2, 3, 4, and 6 from the lean child, respectively; ‘OFF1’, ‘OFF2’, ‘OFF3’, ‘OFF4’, ‘OFF5’, ‘OFF6’, and ‘OFF7’ denote samples 1, 2, 3, 4, 5, 6, and 7 from the obese child, respectively. SCFA denotes short chain fatty acids.

First, based on PCA and hierarchical clustering, we analysed the ability of reaction scores to define consistent clusters that differentiate the different clinical conditions considered. As shown in Fig. 3a, PCA was able to clearly separate lean, allergic, obese, and celiac samples using exclusively reaction scores. Similarly, a hierarchically-clustered heatmap of reaction scores shows good clustering, finding specific patterns for the different individuals (Fig. 3b). Moreover, the heatmap illustrates several differences regarding the normalized reaction activity scores among the different individuals.

Then, we compared the reaction and subsystem activity scores between the disease conditions (allergic to cow’s milk, obese, and celiac) and the control (lean). In particular, at the reaction level, we focused on exchange reactions, which represent the uptake/secretion reactions for microbial metabolites, and they are specifically modelled in genome-scale metabolic reconstructions. The following significant results were obtained (adjusted two-sample Wilcoxon test *P*-value ≤0.05, *ΔΨ* ≥ 0.1): 73 exchange reactions and 22 subsystems in samples from the allergic child, 209 exchange reactions and 33 subsystems in samples from the obese child, and 252 exchange reactions and 20 subsystems in samples from the celiac child (Supplementary Tables S5 and S6).

The different composition of the microbiota in the four analysed children leads to specific metabolic configurations that can be captured by q2-metnet. For example, Fig. 3c shows the boxplot for a top ranked significant subsystem that is under-represented in the samples from the obese child: exchange subsystem of short-chain fatty acids (SCFA), whose supplementation has been associated with reduced body weight (Ecklu-Mensah *et al.* 2023). Figure 3c illustrates activity scores of exchange reactions associated to altered SCFAs, namely acetate (ac), propionate (ppa), iso-butyrate (isobut), and iso-valerate (isoval), which are under-represented in the samples from the obese child with respect to those from the lean child. Similarly, we found supportive literature for top-ranked results in the differential analysis between other clinical conditions and control samples. In the case of celiac disease, for example, we identified a diminished capacity to metabolize isoflavones (Supplementary Fig. S1a), in line with previous studies in the area (Sánchez-Calvo *et al.* 2013). Finally, we observed a reduced capacity of the allergic child to degrade indole metabolites with respect to control samples (Supplementary Fig. S1b), and, interestingly, this family of metabolites protects children against allergic inflammation responses (Zhen *et al.* 2022).

### 3.3 Comparison of *q2-metnet* with other computational methods

In order to assess the effectiveness of q2-metnet in separating properly the samples from the different children discussed in the previous sub-section, we conducted a comparative analysis against other computational methods in the literature. In particular, we predicted the MetaCyc and KEGG metabolic pathway abundances across samples with PICRUSt2, MicFunPred, and Tax4Fun2 algorithms. For each method, we normalized the metabolic pathway abundances and compared them with the subsystem activity scores obtained with *q2-metnet* and mgPipe.

**Figure 4.**
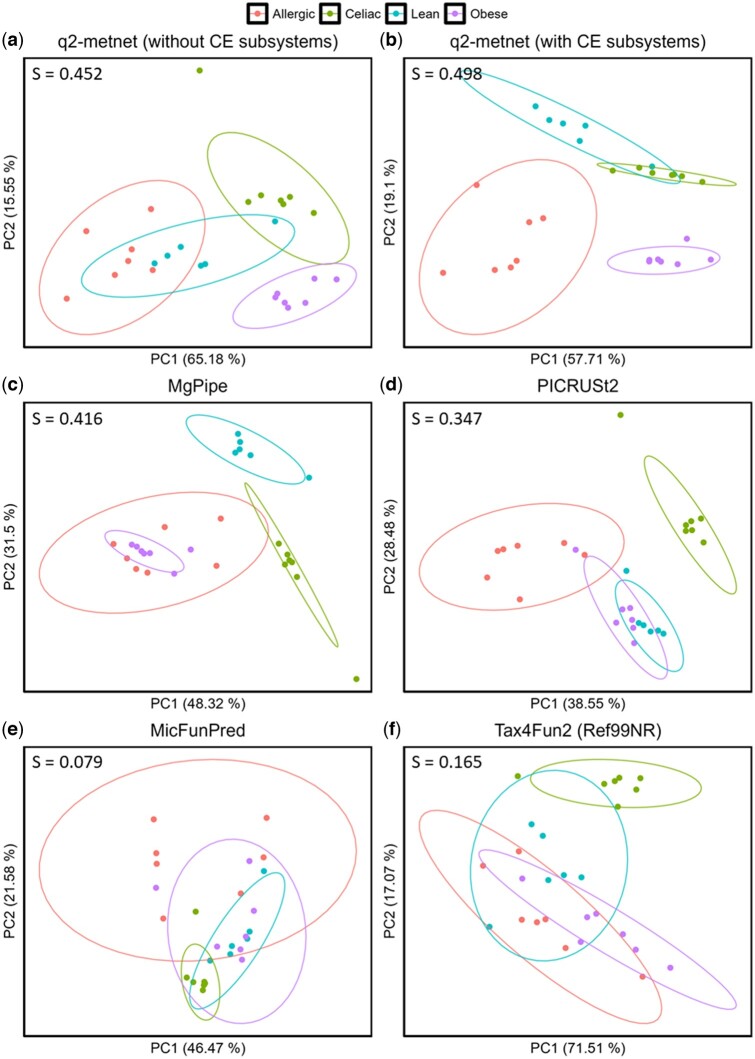
2D visualization of children samples using pathway activity scores of q2-metnet and competing methods in the literature. Principal component analysis (PCA) on pathway activity scores for the four children (lean, allergic to cow’s milk, obese, and celiac) using q2-metnet and AGREDA without nutrient exchange subsystems (a), q2-metnet with nutrient exchange subsystems (b), mgPipe with AGORA (c), PICRUST2 (d), MicFunPred (e) and Tax4Fun2 (f) with the reference dataset of bacterial genomes Ref99NR. The ellipses are generated considering the covariance and standard deviation of the data for each child. *S* values at the top of each plot refer to the Silhouette score values obtained from each PCA.

As shown in Fig. 4, q2-metnet was applied to two different scenarios, namely one considering the default metabolic pathways from AGREDA (Fig. 4a) and one additionally taking into count our CE subsystems (Fig. 4b). In both cases, according to the Silhouette score *S* (0.452 and 0.498, respectively), we were able to more clearly separate the samples from the different children than the rest of algorithms (Fig. 4c–f, *S* <0.42). Only mgPipe and PICRUSt2 obtained a comparable result to that of q2-metnet; however, they were not able to fully distinguish between samples from different clinical conditions. In the case of mgPipe, which is only available for AGORA, the separation between samples from obese and allergic children is unclear. The same occurs in PICRUSt2 for samples of obese and lean children. In the case of q2-metnet, the samples from the allergic to cow’s milk and lean children seem to slightly overlap in Fig. 4a, whereas they significantly differ from the rest of samples. Interestingly, q2-metnet was able to distinguish between these two groups of samples when CE exchange subsystems were considered (Fig. 4b). The different dietary intake of allergic children that do not consume cow’s milk may have resulted in an altered abundance of nutrient exchange subsystems in their gut microbiota. In addition, q2-metnet obtained better separation with AGREDA than in AGORA and AGORA2 (Supplementary Fig. S2), which reflects the relevance of the subsystems related with phenolic compounds, more accurately defined in AGREDA.

In order to evaluate the performance of q2-metnet in a more complex scenario, we additionally analysed 16S rRNA gene sequencing data of 1024 individuals from the BFGFP (Falony *et al.* 2016). In particular, we classified individuals into ‘obese’ and ‘normal weight’ according to their Body Mass Index (BMI), namely BMI >30 for obese individuals and 18 < BMI <25 for normal weight individuals. The taxonomic assignment of samples was done using the same procedure as in the previous sub-section. Then, using q2-metnet, we compared the subsystem scores between 134 obese and 568 normal weight individuals, obtaining 7 out of 172 subsystems with significant differences among groups (adjusted two-sample Wilcoxon test *P*-value ≤.05, Supplementary Table S7). Interestingly, we found three CE subsystems with significant alterations: indole (increased in obese individuals), purines (decreased in obese individuals), and benzoic alcohol (increased in obese individuals). For indole and purine metabolites, we found supporting literature that agrees with our predictions (Calvani *et al.* 2010, Zhang and Dang 2022). Among the rest of the subsystems, it is remarkable the alterations in vitamin B2 metabolism, which has been found to be decreased in obese individuals (Mazur-Bialy and Pocheć 2016), in line with the outcome from q2-metnet.

We conducted the same analysis with mgPipe and PICRUSt2 for the BFGFP cohort. In the case of mgPipe, we found a larger number of alterations, namely 24 out of 150 subsystems showed significant differences among groups (adjusted two-sample Wilcoxon test *P*-value ≤.05, Supplementary Table S7). This result shows that the differences between q2-metnet and mgPipe in the subsystem score calculation strategy have a relevant impact in the outcome. In fact, we found a negative correlation between the *P*-values of the common subsystems between mgPipe and q2-metnet (Pearson correlation level = −0.1). In addition, mgPipe deemed 1 out of the 4 subsystems in common with q2-metnet as significant. With respect to PICRUSt2, we identified significant changes in 9 out of 374 MetaCyc pathways, which have little association with the results of q2-metnet and mgPipe. Overall, these results show that q2-metnet obtains results that are complementary with other approaches in the literature.

## 4 Discussion

High-quality metabolic reconstructions of the human gut microbiota, such as AGORA or AGREDA, have emerged in the last years. In general, they have been integrated into constraints-based models for predicting the synthesis of key bioactive metabolites, the degradation of dietary compounds and drug metabolism, and bacteria–bacteria interactions, among others. However, their integration with standard bioinformatic tools for the study of human microbiome data has not been systematically addressed. In this article, we cover this gap and integrate AGORA and AGREDA with QIIME2, a powerful microbiome analysis package.

QIIME2 enables the development of plugins to provide new functionalities. Here, we present q2-metnet, a QIIME2 plugin that enables the contextualization of 16S rRNA data into AGORA and AGREDA. In particular, based on taxonomic abundances, q2-metnet determines normalized activity scores for the reactions and subsystems involved in the selected metabolic reconstruction. Using these scores, q2-metnet allows the user to conduct differential activity analysis for reactions and subsystems, as well as exploratory analysis using PCA and hierarchical clustering. We illustrate the capabilities of q2-metnet in two different datasets, finding clear metabolic alterations in both cases at the reaction and subsystem activity level.

With respect to previous approaches in the literature, q2-metnet follows a reaction-centric strategy that allows us to capture different and complementary metabolic features to the ones obtained with gene-centric approaches, such as PICRUSt2, MicFunPred, and Tax4Fun2. In addition, q2-metnet presents clear advantages over mgPipe, a previous reaction-centric approach in the literature, which is not available in QIIME2 and it is currently restricted to AGORA. Moreover, q2-metnet and mgPipe implement different strategies at the methodological level, particularly in the way reaction activity scores are integrated into subsystems, which has a tremendous impact in the final outcome, as it is observed in the case study of the BFGFP cohort.

In the case-study shown in Fig. 4, q2-metnet presents a better separation of samples from children with different clinical conditions, which supports that our computational methodology constitutes a relevant alternative to conventional algorithms based on metagenome prediction and mgPipe. In addition, in the analysis of the BFGFP cohort, q2-metnet identified seven metabolic subsystems significantly altered in samples from obese individuals. We found with good agreement in the literature for four out of these seven subsystems. Notably, only one of these seven subsystems were captured by other approaches, which illustrate the high level of complementary of q2-metnet with the rest of methods.

Overall, we are confident that the q2-metnet package will improve the analysis of the metabolic capabilities of the human gut microbiota, enabling the extraction of testable differences among the conditions analysed.

## Supplementary Material

btae455_Supplementary_Data

## Data Availability

The 16S rRNA sequencing data for children under different clinical conditions are available at https://www.ebi.ac.uk/ena/browser/home under accession code PRJEB40603. 16S rRNA sequencing data from the Belgian Flemish Gut Flora Project (BFGFP) cohort is available at https://ega-archive.org/ under the accession code EGAD00001001936.
