# Supplementary material for: q2-metnet: QIIME2 package to analyse 16S rRNA data via high-quality metabolic reconstructions of the human gut microbiota
Source: Bioinformatics. 2024 Jul 17;40(11):btae455. doi: 10.1093/bioinformatics/btae455 (PMC11561042; doi:10.1093/bioinformatics/btae455)
Supplement: btae455_Supplementary_Data [file btae455_supplementary_data.zip › Supplementary Information.docx]

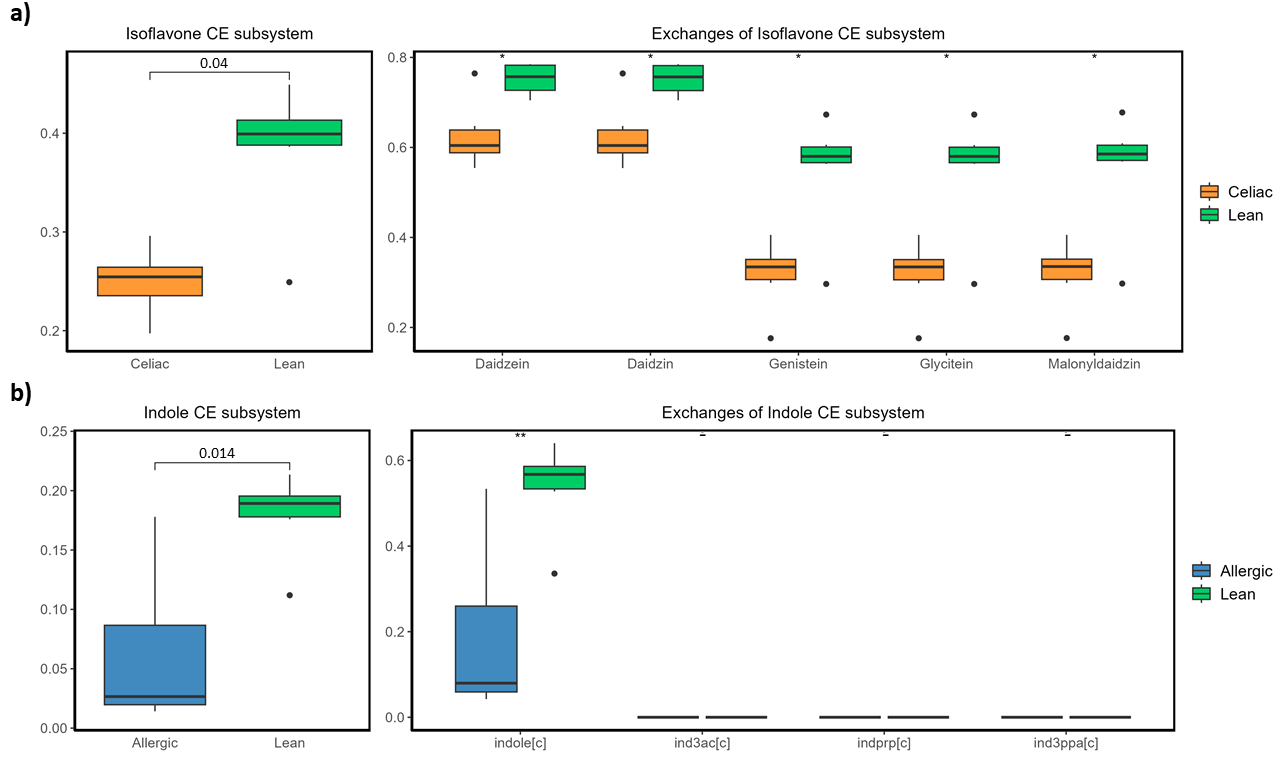


**Supplementary Figure 1: Activity score of top-ranked subsystem among clinical conditions. a)** Boxplot of activity score of isoflavone CE subsystem and its corresponding exchange reactions in celiac and lean children. **b)** Boxplot of activity score of indole CE subsystem and its corresponding exchange reactions in allergic to cow´s milk and lean children. P-value was calculated using two-sample Wilcoxon test and adjusted with False Discovery Rate approach. Abbreviations: *ind3ac* (Indole-3-acetate), *indprp* (Indolepropionate), *ind3ppa* (Indole-3-propionate).


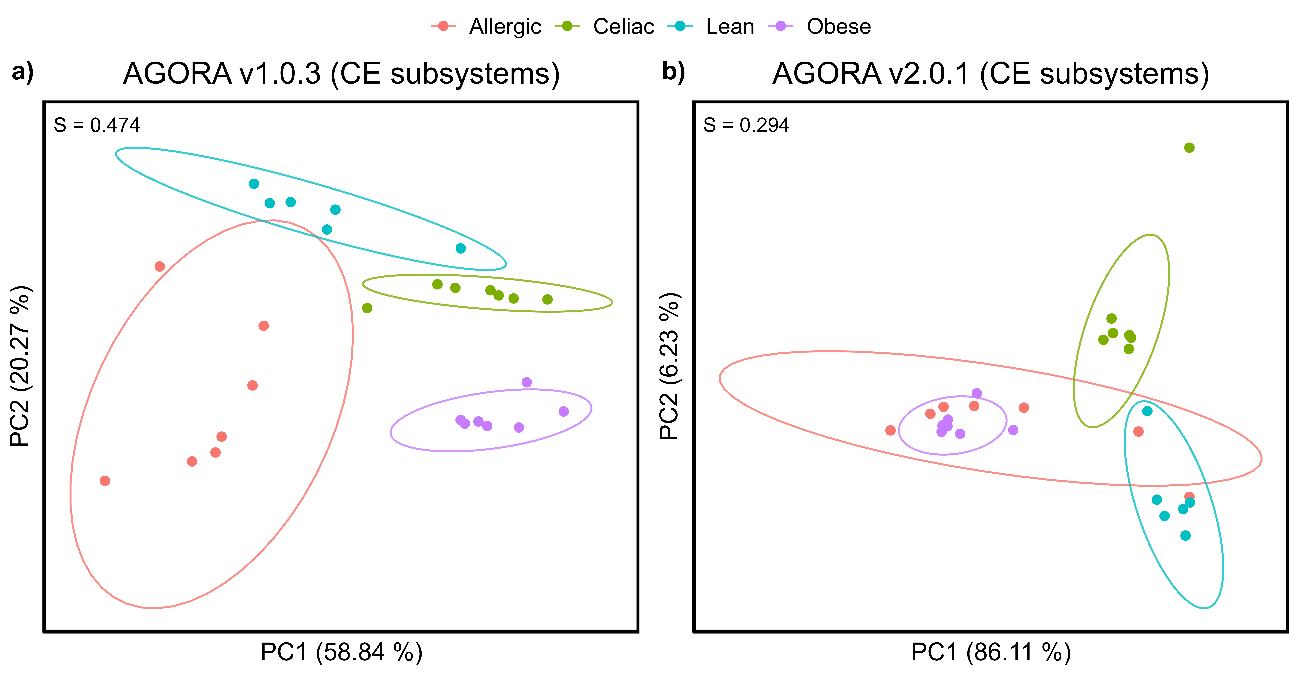


**Supplementary Figure 2: 2D visualization of children samples using pathway activity scores of *q2-metnet* and competing methods in the literature.** Principal Component Analysis (PCA) on pathway activity scores for the four children (lean, allergic to cow’s milk, obese and celiac) using *q2-metnet* and AGORA 1.0.3 with nutrient exchange subsystems (**a**) and *q2-metnet* and AGORA2 2.0.1 with nutrient exchange subsystems (**b**). The ellipses are generated considering the covariance and standard deviation of the data for each child. S values at the top of each plot refer to the Silhouette score values obtained from each PCA.
